# Supplementary material for: Giant transverse magnetic fluctuations at the edge of re-entrant superconductivity in UTe2
Source: Nat Commun. 2026 Apr 29;17:3742. doi: 10.1038/s41467-026-71899-7 (PMC13128824; doi:10.1038/s41467-026-71899-7)
Supplement: Supplementary file 1 — Supplementary Information [file 41467_2026_71899_MOESM1_ESM.pdf]

Supplementary information for "Giant transverse magnetic fluctuations at the edge of re-entrant superconductivity in UTe_2"

I. CRYSTAL AXES & ALIGNMENT

Accurate measurements of the magnetotropic susceptibility $k = \partial^2 F/\partial\theta^2$ are sensitive to the alignment of the crystallographic axes with respect to the external magnetic field. In order to identify the crystallographic axes, we performed X-ray scattering using a commercial Laue detector from Photonic Science. Figure 1 shows the a- and b-axes of the orthorhombic structure of UTe_2.

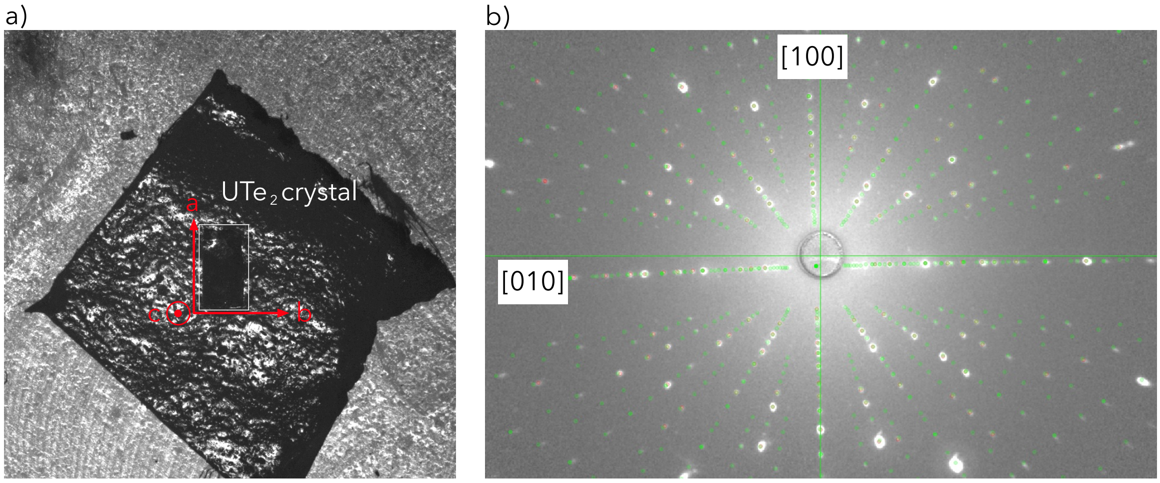

FIG. 1. **Laue diffraction on Sample #2** The crystal was mounted with the c-axis perpendicular to the surface of a scanning electron microscopy (SEM) stub. An example of the diffraction spectra showing the alignment of the a-axis and b-axis relative to the sample.

After identifying the a- and b-axes using Laue, the axes were marked on the mounting stub and were kept track of when cutting small pieces off for Sample #2 and Sample #3, as well as while cutting sample #1 in the FIB.

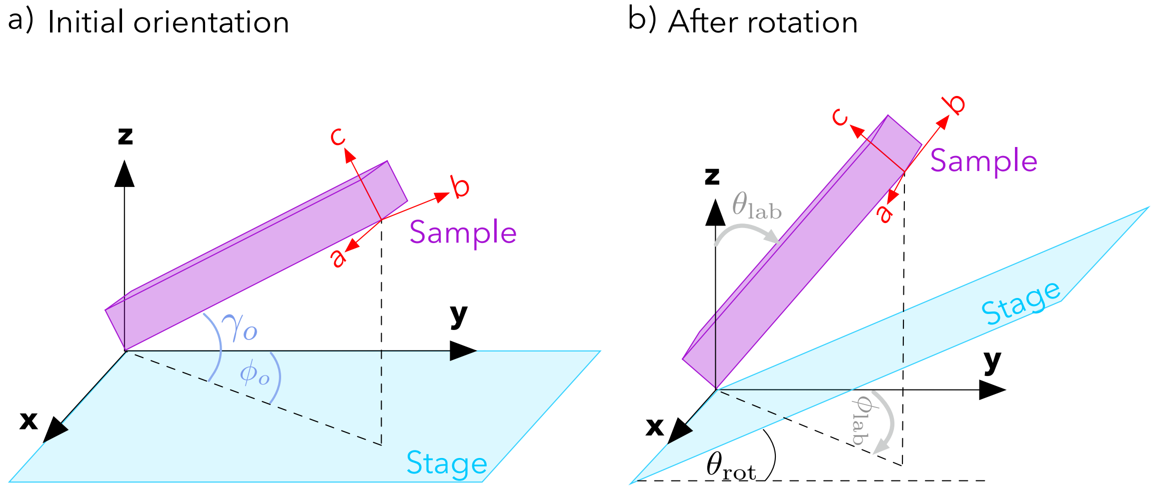

FIG. 2. **Schematic of the sample alignment on the rotation stage.** The axes $\mathbf{x}, \mathbf{y}, \mathbf{z}$ denote the lab reference frame, with magnetic field along the $\mathbf{z}$-axis. a) Starting from the crystal axes a, b, and c aligned with $\mathbf{x}$, $\mathbf{y}$, and $\mathbf{z}$, respectively, γ_o defines offset of the sample around the $\mathbf{x}$-axis, and ϕ_o defines the offset around the $\mathbf{z}$ axis. Note that the offset γ_o is applied before the offset ϕ_o b) Sample orientation after the stage is rotated by $\theta_{\rm rot}$ around the $\mathbf{x}$-axis. $\theta_{\rm lab}$ and $\phi_{\rm lab}$ describe the orientation of the sample axes with respect to the magnetic field in the lab frame, as given by Equation 4.

In our experiment, we consider that the sample is mounted with a small misalignment (exaggerated in Figure 2), and is fixed in place onto a rotation stage. Starting from the crystal axes a, b, and c aligned with $\mathbf{x}$, $\mathbf{y}$, and $\mathbf{z}$, respectively, the sample is first offset around the $\mathbf{x}$ axis by an angle γ_0 (the axes and angles are defined in Figure 2). Next, the sample is offset by an angle ϕ_0 around the $\mathbf{z}$ axis. The orientation of the sample on the stage is then

$$\mathbf{m}_{\rm s} = \big(\sin(90^\circ - \gamma_0)\cos(90^\circ - \phi_0),\ \sin(90^\circ - \gamma_0)\sin(90^\circ - \phi_0),\ \cos(90^\circ - \gamma_0)\big). \quad (1)$$

In our experiment, the magnetic field is always applied along the **z** axis. The initial orientation of the stage is such that its normal is parallel to the **z**-axis (see Figure 2). Before for each measurement, we rotate the stage by an angle θ_{rot} around the **x**-axis. The sample orientation in the lab frame after each rotation is then

$$\mathbf{m}_{\mathrm{lab}} = \begin{pmatrix} 1 & 0 & 0 \\ 0 & \cos(\theta_{\mathrm{rot}}) & -\sin(\theta_{\mathrm{rot}}) \\ 0 & \sin(\theta_{\mathrm{rot}}) & \cos(\theta_{\mathrm{rot}}) \end{pmatrix} \cdot \mathbf{m}_{\mathrm{s}}, \tag{2}$$

$$\mathbf{m}_{\mathrm{lab}} = (\sin\theta' \cos\phi',\ \cos\theta_{\mathrm{rot}} \sin\theta' \sin\phi' - \sin\theta_{\mathrm{rot}} \cos\theta',\ \sin\theta_{\mathrm{rot}} \sin\theta' \sin\phi' + \cos\theta_{\mathrm{rot}} \cos\theta'), \tag{3}$$

where $\theta' = (90° - \gamma_o)$ and $\phi' = (90° - \phi_o)$. Therefore θ_{lab} and ϕ_{lab} of the sample are

$$\begin{aligned} \theta_{\mathrm{lab}}\,[\theta_{\mathrm{rot}}, \gamma_o, \phi_o] &= \arccos\big(\sin\theta_{\mathrm{rot}} \sin\theta' \sin\phi' + \cos\theta_{\mathrm{rot}} \cos\theta'\big)\big), \\ \phi_{\mathrm{lab}}\,[\theta_{\mathrm{rot}}, \gamma_o, \phi_o] &= \arctan\big(\cos\theta_{\mathrm{rot}} \sin\theta' \sin\phi' - \sin\theta_{\mathrm{rot}} \cos\theta', \sin\theta' \cos\phi'\big). \end{aligned} \tag{4}$$

Equation 4 provides the angles θ_{lab} and ϕ_{lab} with respect to the magnetic field of the sample given the initial offsets γ_o and ϕ_o.

Based on Lewin et al. [2], the critical field H_{m} of the metamagnetic transition has the following dependence on the polar θ and the azimuthal ϕ orientation of the magnetic field within the crystal

$$H_{\mathrm{m}} = \frac{H_{\mathrm{m}}^b}{\cos(90° - \theta)} + \alpha_2 \sin^2(\phi) + \alpha_4 \sin^4(\phi). \tag{5}$$

θ is the angle between the magnetic field and the c-axis and ϕ is the angle between the magnetic field and the b-axis. H_{m}^b denotes the value of H_{m} for $\mathbf{B} \parallel b$-axis. We adopt the constants reported by Lewin et al. [2]: $H_{\mathrm{m}}^b = 34$ T, $\alpha_2 = 95$ T, and $\alpha_4 = 1934$ T.

Now, we introduce the misalignments θ_{lab} and ϕ_{lab} into Equation 5 to find γ_o and ϕ_o using our experimental data. From our bc-plane data, we define H_{m} as the point where the minimum in k occurs right before the sharp increase onsets with increasing field (Figure 3).

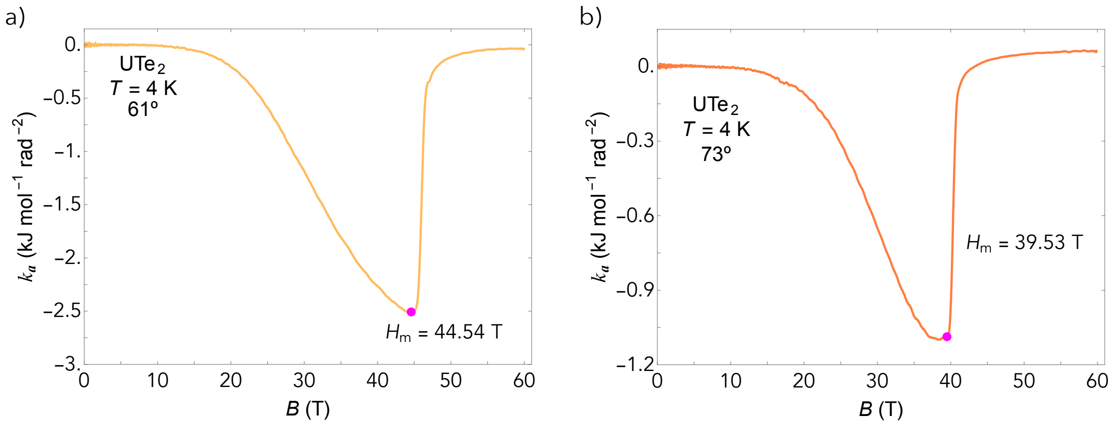

FIG. 3. **The magnetotropic susceptibility k in UTe_2 at $T = 4$ K for two angles in the bc-plane: a) $\theta = 61°$ and b) $\theta = 73°$.** This data is collected from Sample #1 and corresponds to the data shown in the main text. The magenta dots indicate how we selected the metamagnetic transition.

In Figure 3, we found $H_{\mathrm{m}} = 44.5$ T at $\theta = 61°$, which corresponds to a stage rotation of $\theta_{\mathrm{rot}} = 90 - \theta = 29°$. Similarly, from Figure 3, $H_m = 39.53$ T at $\theta = 73°$, corresponding to a stage rotation of $\theta_{\mathrm{rot}} = 17°$. Then, using Equation 4 and Equation 5, we can build the following system of equations:

$$\begin{aligned} 44.54\ \mathrm{T} = &\frac{34\ \mathrm{T}}{\cos(90° - \theta_{\mathrm{lab}}[29°, \gamma_0, \phi_0])} + 95\ \mathrm{T} \sin^2(\phi_{\mathrm{lab}}[29°, \gamma_0, \phi_0]) \\ &+ 1934\mathrm{T}\ \sin^4(\phi_{\mathrm{lab}}[29°, \gamma_0, \phi_0]), \end{aligned} \tag{6}$$

$$\begin{aligned} 39.53\ \mathrm{T} = &\frac{34\ \mathrm{T}}{\cos(90° - \theta_{\mathrm{lab}}[17°, \gamma_0, \phi_0])} + 95\ \mathrm{T} \sin^2(\phi_{\mathrm{lab}}[17°, \gamma_0, \phi_0]) \\ &+ 1934\ \mathrm{T}\ \sin^4(\phi_{\mathrm{lab}}[17°, \gamma_0, \phi_0]). \end{aligned} \tag{7}$$

Solving this system of equations, we find $\gamma_o = 3.86°$ and $\phi_o = 8.16°$ for Sample #1 (the data presented in the main text).

Once we determine γ_0 and ϕ_0, it is possible to calculate $\theta_{\rm lab}$ and $\phi_{\rm lab}$ of the sample for every measurement at different angles, and then estimate $H_{\rm m}$ using Equation 5. Figure 4 shows a comparison between the experimental values $H_{\rm m\_exp}$ and the estimated ones $H_{\rm m\_est}$, where we observe a fairly good agreement. This analysis shows that the actual misalignments of our sample are well represented by the γ_o and ϕ_o determined using Equation 5.

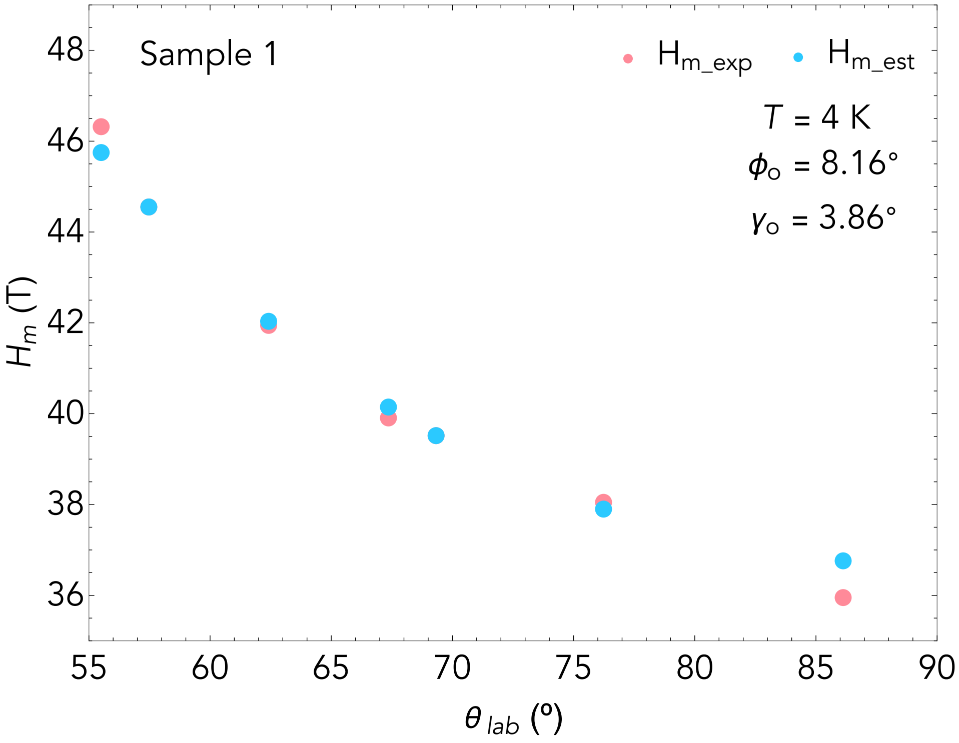

FIG. 4. **Experimental and estimated metamagnetic transitions in Sample #1.** Comparison of the measured metamagnetic transition $H_{\rm m\_exp}$ and the estimated metamagnetic transition $H_{\rm m\_est}$ in UTe_2 versus the polar angle $\theta_{\rm lab}$ — the corrected polar angle between the c-axis and the magnetic field taking into account the misalignments.

A similar procedure was employed to determine the misalignment of Sample #3, again restricting the analysis to samples where we have bc-plane data because we rely on the position of the metamagnetic transitions to guide us. For Sample #3, we find $\gamma_o = -9.5°$ and $\phi_o = 12.0°$ (Figure 5).

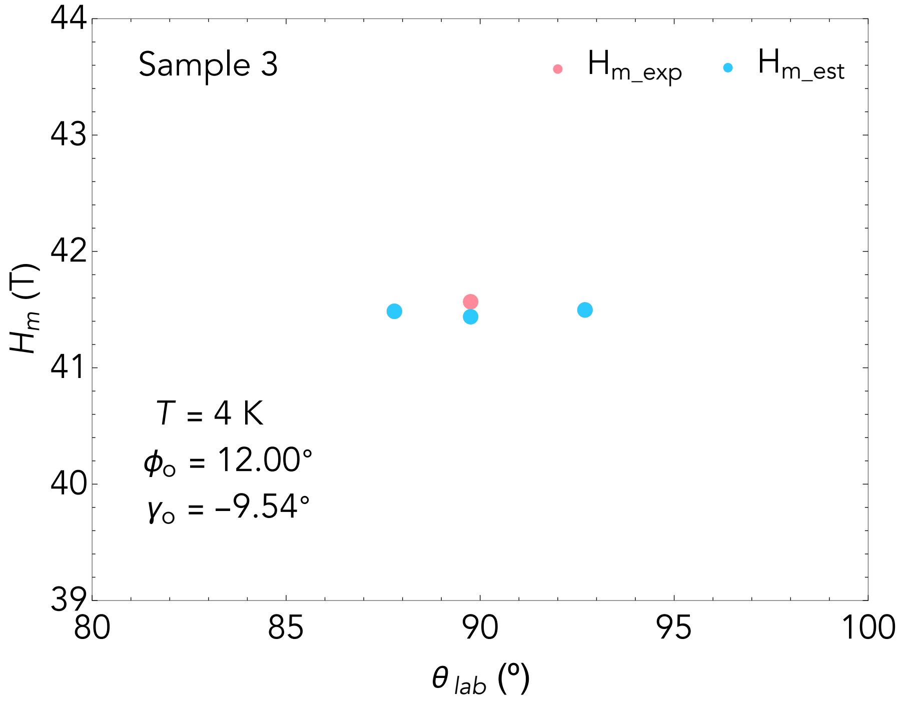

FIG. 5. **Experimental and estimated metamagnetic transitions in Sample #3.** Comparison of the experimental and estimated metamagnetic transitions, $H_{\mathrm{m\_exp}}$ and $H_{\mathrm{m\_est}}$, for Sample #3. The misalignments determined are larger than that found for Sample #1.

Figure 6 shows the metamagnetic transition H_m as determined by Lewin et al. [2], extrapolated as the purple surface as a function of θ and ϕ. This surface was created using Equation 5. The gray plane that intersects the surface corresponds to the case in which the sample is perfectly aligned in the bc-plane, showing a metamagnetic transition at 34 T for field along the b-axis ($\theta = 90°$). Taking into account the misalignments γ_o and ϕ_o, the additional colored planes represent how the measurement planes cross the metamagnetic phase boundary for samples #1 and #3. The data points correspond to the experimentally-determined values of H_m. Note that the range in ϕ suggests that the misalignments are within roughly 10°—the precise misalignment angles are given in Table I.

Note that the critical endpoints found by Wu et al. [4] are not included in the extrapolation of the metamagnetic phase shown in Figure 6. If the critical endpoints were included, the purple surface would be terminated abruptly according to the angle dependence of the line of critical endpoints. Based on our measurements of Sample #1, for example, in the main text, the purple surface would not extend past 59° or 46 T, where the metamagnetic transition is terminated by the critical endpoint [4].

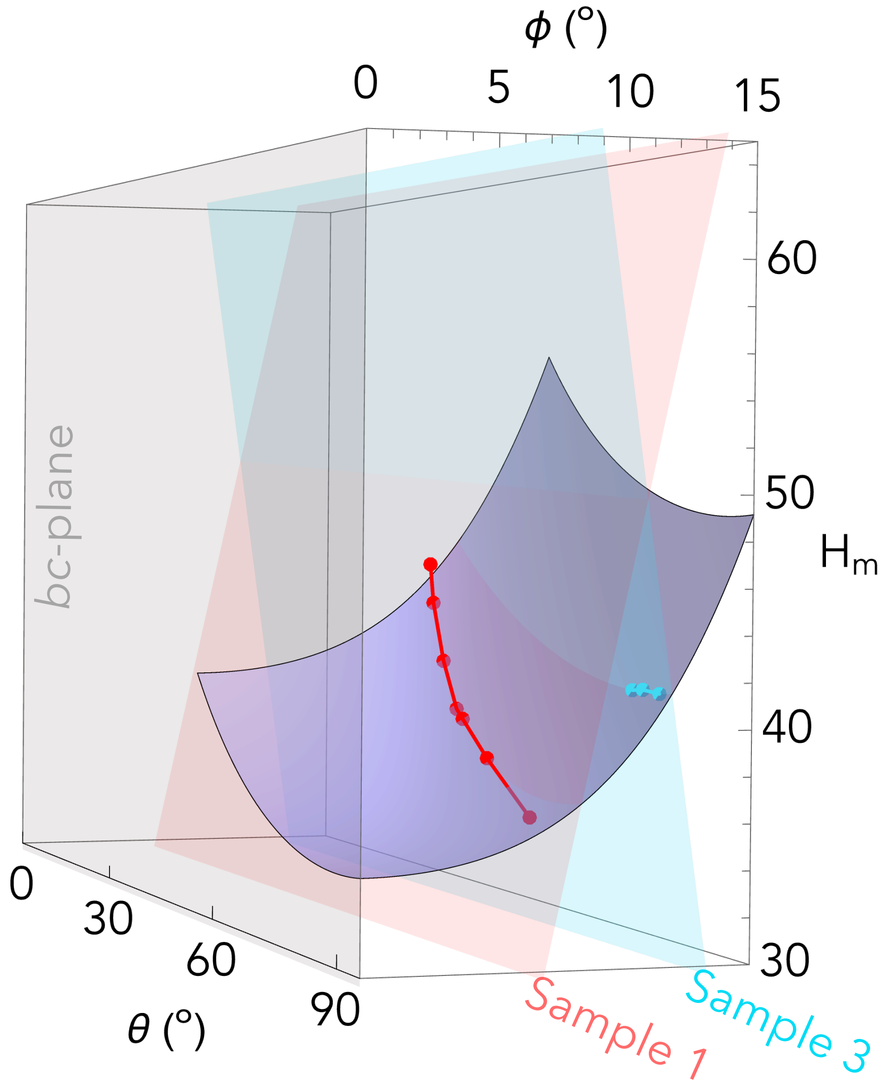

FIG. 6. **Predicted (surfaces) and measured (points) metamagnetic transitions.** The metamagnetic transition determined by Lewin et al. [2], extrapolated in field-angle space (purple surface). ϕ represents rotation of magnetic field from the b-axis towards a. θ corresponds to rotation in the bc-plane with field along the c-axis defined as $\theta = 0$. The black plane shows the dependence of H_{m} upon field rotation in the bc-plane (*i.e.* if the sample was perfectly aligned). The red and blue planes corresponds to the actual measurement planes for Samples #1 and #3, respectively.

TABLE I. Angle misalignment of the measured samples. ϕ_0 corresponds to the misalignment of the sample with respect to the b axis in the ab plane. γ_o describes the tilt of the sample with respect to the the cantilever.

Sample	ϕ_o [°]	γ_o [°]
1	8.16	3.86
3	12.00	-9.54

II. SAMPLE PREPARATION

In highly-anisotropic materials, magnetotropic signal sizes vary substantially with magnetic field orientation. In order to achieve an appropriate signal size in the magnetotropic measurements for magnetic field rotated throughout these highly-anisotropic planes (i.e. to avoid breaking the cantilever), we need to prepare small samples. In total, we prepared 3 samples from 2 different bulk pieces (separate single crystals from the same batch). Two samples were cut by hand, and for one sample (Sample #1 used in the main text) we used the Helios G4 xenon plasma focused-ion beam (FIB). Use of the FIB allowed for better sample alignment. The overall trends in the high-field results, from both the *ac*- and *bc*-plane measurement, are consistent between all three samples, with slight differences due to misalignment and the high sensitivity of UTe_2 to field angle. Sample information is summarized in Table II below.

Once the crystal axes were identified with respect to the macroscopic crystal, the sample was mounted with the *b*-axis perpendicular to the surface of the sample holder and placed into the FIB chamber (Figure 7). Samples were cut

TABLE II. Summary of the samples measured and their relevant parameters

Sample	Bulk	Type	Volume [μm^3]	mol of U	k for 1 Hz [J/mol]
1	A	FIB'ed	72,914	1.36×10^{-9}	0.77
2	B	Hand cut	456,624	8.53×10^{-9}	0.54
3	B	Hand cut	568,620	1.06×10^{-8}	0.43

from the bulk single crystal using small currents (4-15 nA) at 20 kV to minimize sample heating and surface damage.

After cutting and polishing the sides of the samples using the ion beam, the samples were left hanging from the bulk crystal by a $\sim 1 \times 1$ μm^2 bridge. Under an optical microscope, Sample #1 was manually disconnected from the bulk piece using an eyelash and a kapton needle. Before transferring the sample to the silicon microcantilever for measurements, a small drop of Apiezon L grease was placed on the lever to ensure that magnetic torque on the sample during measurements does not displace it. After measurements, we check that the placement of the sample on the lever remained intact.

Sample #2 and Sample #3 were cut using a razor blade from a different bulk sample than Sample #1. Before cutting the crystal, we used Laue to confirm the crystal axes. We then painted each face a different color in order to correlate the crystal axes in the cut piece with those from the Laue results.

For both measurement planes, the c-axis of the sample was mounted perpendicular to the surface of the lever and the a- or b-axis was mounted parallel to the long axis of the lever, depending upon the plane of anisotropy to be measured (ac- or bc-plane). After measurements in the first plane, the sample was rotated under an optical microscope by hand by $\sim 90°$.

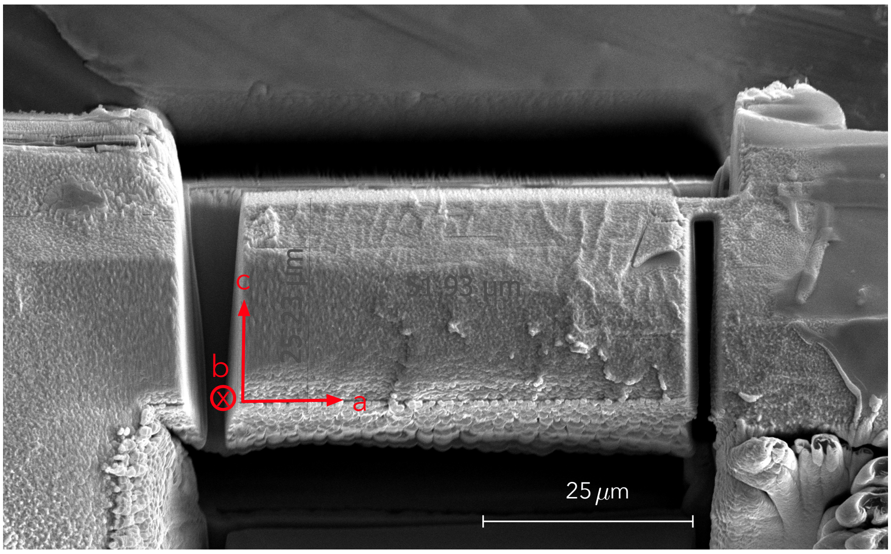

FIG. 7. **FIB'ed UTe_2 Sample #1** The dimensions are $\sim 52 \times 66 \times 25$ μm^3.

II.1. Reproducibility

We performed high-field magnetotropic measurements on two different bulk crystals: Sample #1 was FIB'ed and Samples #2 and #3 were cut from a bulk piece using a razor blade. The data included in the main text was taken from Sample #1. We also acquired data in the ac- and bc-planes for Sample #2 and Sample #3. We find that the data taken across all samples is consistent; in particular, the large decrease in magnetotropic susceptibility, indicating a large increase in transverse susceptibility in the bc plane, is consistent between samples.

Figure 8 shows the magnetotropic susceptibility taken at temperatures below T_c for field aligned along each of the crystallographic directions in two different samples. The red curve shows data taken at the lowest temperature of 625 mK. The overall features observed in Figure 2 c&d at $T = 4$ K in the manuscript are reproduced here in Figure 8. The key consistencies are: i) the overall magnitude of the signal for all field configurations (*i.e.* a small response for $B||a$ and $B||b$ up to 60 T) ii) the onset of softening near 20 T and the metamagnetic transition near 35 T for $B||b$, and iii) the large softening that onsets near 20 T for $B||c$. This also demonstrates that the transverse softening persists down to low temperatures.

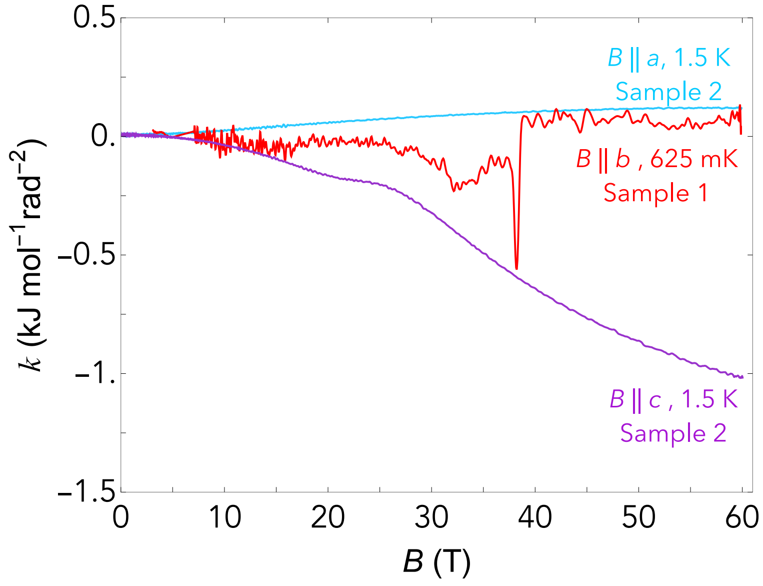

FIG. 8. **Magnetotropic measurements in the superconducting state** At temperatures below T_c (T_c = 1.85 K in our samples), the magnetotropic susceptibility versus magnetic field for the three crystallographic directions. On Sample #1, k was measured for $B||b$ at T = 625 mK. The large increase in noise is due to the reduction in vibration damping because the 4He bath is superfluid at these temperatures. The onset of the magnetotropic softening – small at this angle – is visible as a downturn near 25 T as a precursor to the metamagnetic transition near 35 T. The large magnetotropic softening near 20 T in Sample #2 at 1.5 K reproduces the main finding in the manuscript observed in the bc-plane for Sample #1. The blue curve shows k for $B||a$, also for Sample #2, which exhibits an essentially flat response up to 60 T – again consistent with Figure 2c in the manuscript.

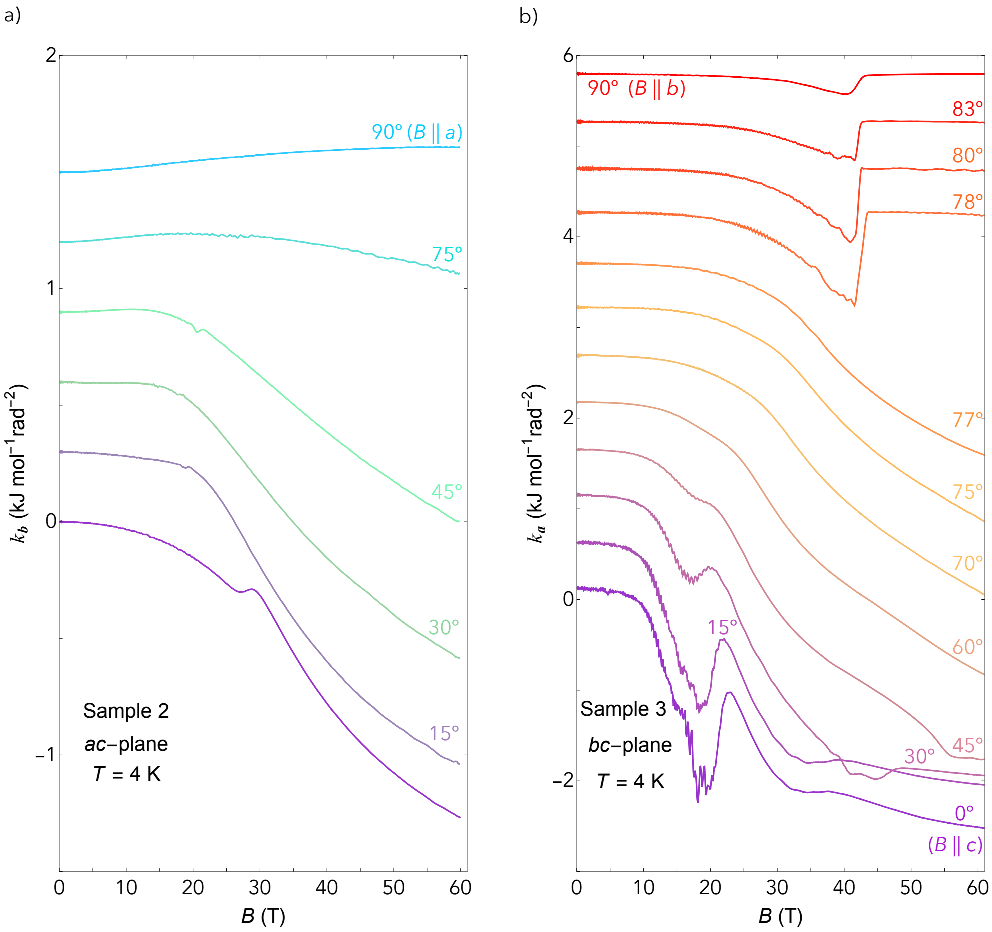

FIG. 9. **Magnetotropic susceptibility in the ac- and bc-planes** a) The ac-plane of Sample #2 was rotated with respect to field for several 60 T magnetic field pulses. A large decrease in the magnetotropic susceptibility is observed, similar in magnitude to that observed in Figure 2 of the main text. b) For rotation in the bc-plane, a large decrease in the magnetotropic susceptibility is again observed in a region consistent with the data shown in the main text. Between $\theta = 77°$ and $\theta = 78°$, the softening is cut off by the metamagnetic phase boundary that extends from $B||b$. The difference in $B^\star$ between this data and the data in the main text is due to a great degree of sample misalignment for this sample. However, the features as a function of angle are consistent with other studies if we assume an angle offset [1]. A large discontinuity indicating a possible second-order phase transition—possibly the $B^\star$ line from the second-order endpoint of the metamagnetic transition—is observed near 20 T in Sample #3.

Figure 9 demonstrates that the main findings observed in the magnetotropic susceptibility in Sample #1 (a FIB'ed sample) of the manuscript are reproduced here for two different samples. In particular, we find 1) a large softening observed at ∼20 T for a wide range of angles in both the ac- and bc-plane and 2) a critical endpoint to the line of first-order metamagnetic transitions in the bc-plane. In Sample #3, we observe that the softening onsets at a slightly lower field of ∼10 T compared to ∼20 T in Sample #1 in the manuscript. We also observe a large discontinuity in the middle of the field range over which the softening occurs for several angles near the c-axis, for measurements both in the ac- and bc-planes. This jump down followed by a peak in the magnetotropic susceptibility upon approaching 20 T is characteristic of a second-order phase transition, which was not observed in Sample #1. We attribute this difference to slight misalignments of the bc-plane on the lever for both samples; we believe in Sample #1, we precess around the phase transition observed in Sample #3. This is in agreement with the fact that the metamagnetic transition and the critical endpoint also occur at slightly different angles in both experiments. Alignment of our samples, which are only 100's of nanograms, is a technical challenge. As all features in UTe_2 are highly-anisotropic, one expects that slight misalignments may lead to variation in the positions of features observed with angle. Assuming ≈ 10° offsets, our data are consistent with that of Lewin et al. [1].

III. CALIBRATION

In order to convert the measured frequency into the correct magnetotropic susceptibility units (J/mol/rad^2), we use the linear response regime $M_i = \chi_{ij} H_j$. Here, the frequency shift, which is directly proportional to the magnetotropic susceptibility, follows a characteristic field- and angle-dependence $k = (\chi_i - \chi_j)\cos 2\theta B^2$, where i, j describe the principal magnetic axes of the crystal in the plane of oscillation. Figure 10 shows the measured frequency shift vs B after subtracting their respective zero-field frequencies. After subtraction, a quadratic fit to the low-field (from ∼1-7 T) data gives the proportionality factor of the magnetic anisotropy.

A plot of the coefficient to the quadratic behavior in Figure 10a versus angle yields the anisotropy in the magnetic

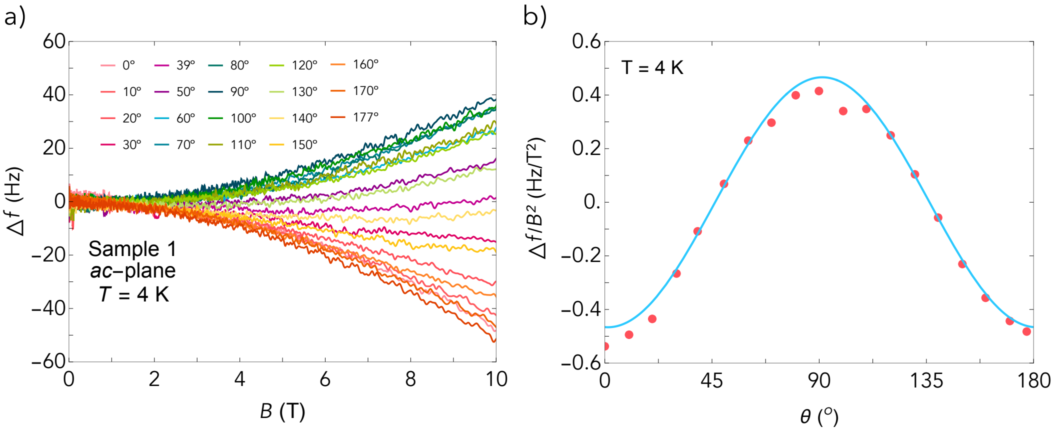

FIG. 10. **Magnetotropic susceptibility at low fields on Sample #1 in the** ac**-plane** a) At $T = 4$ K, the measured frequency shift after subtracting the zero-field frequency versus magnetic field up to 10 T at various field orientations. The frequency shift is proportional to the magnetotropic susceptibility, which has a quadratic field dependence at low fields due to a linear-in-field magnetization. b) The coefficient to the quadratic-in-field dependence of the magnetotropic susceptibility follows a cos2θ dependence and is proportional to the anisotropic magnetic susceptibility in the plane of vibration ($\chi_i - \chi_j$).

susceptibility, $\chi_i - \chi_j$ (Figure 10b). At $\theta = 0°$, the amplitude is $\sim$ -0.47 Hz/T^2. The anisotropic susceptibility obtained through magnetic susceptibility measurements for field aligned along the a- and c-axes is $\chi_a - \chi_c = 0.36$ J/T^2/mol [3]. This can be equated with the amplitude of angle-dependent k measurements

$$0.47 \left[\frac{\text{Hz}}{\text{T}^2}\right] \rightarrow 0.36 \left[\frac{\text{J}}{\text{T}^2\ \text{mol}}\right] \tag{8}$$

Therefore, the conversion factor applied to the magnetotropic measurements for Sample #1 is 1 Hz $\rightarrow$ 0.77 J/mol/rad^2.

Because the anisotropy in the bc-plane at low fields is so small, the same calibration factor was applied to the bc-plane data for unit conversion. This is valid because we use the same sample on the same cantilever, and thus the calibration factor—related to the bending stiffness of the cantilever—is the same.

The magnetotropic susceptibility divided by magnetic field can be represented in units of magnetization. Based on the volume of the sample, the unit cell volume, and the fact that each (conventional) unit cell has 4 uranium atoms, we estimate 1.36×10^{-9} moles of uranium in Sample #1. With $\mu_B = 9.2 \times 10^{-24}$ J/T, this allows for conversion of the frequency shift into units of μ_B per U:

$$1 \left[\frac{\text{Hz}}{\text{T}}\right] \rightarrow 0.14 \left[\frac{\mu_B}{\text{U}}\right]. \tag{9}$$

[1] Sylvia K. Lewin, Josephine J. Yu, Corey E. Frank, David Graf, Patrick Chen, Sheng Ran, Yun Suk Eo, Johnpierre Paglione, S. Raghu, and Nicholas P. Butch. Field-angle evolution of the superconducting and magnetic phases of UTe2 around the b axis. *Physical Review B*, 110(18):184520, 2024. ISSN 2469-9950. doi:10.1103/physrevb.110.184520.

[2] Sylvia K. Lewin, Peter Czajka, Corey E. Frank, Gicela Saucedo Salas, G. Timothy Noe II, Hyeok Yoon, Yun Suk Eo, Johnpierre Paglione, Andriy H. Nevidomskyy, John Singleton, and Nicholas P. Butch. High-field superconducting halo in UTe2. *Science*, 389(6759):512–515, 2025. ISSN 0036-8075. doi:10.1126/science.adn7673.

[3] Priscila F. S. Rosa, Ashley Weiland, Shannon S. Fender, Brian L. Scott, Filip Ronning, Joe D. Thompson, Eric D. Bauer, and Sean M. Thomas. Single thermodynamic transition at 2 K in superconducting UTe2 single crystals. *Communications Materials*, 3(1):33, 2022. doi:10.1038/s43246-022-00254-2.

[4] Z. Wu, T. I. Weinberger, A. J. Hickey, D. V. Chichinadze, D. Shaffer, A. Cabala, H. Chen, M. Long, T. J. Brumm, W. Xie, Y. Ling, Z. Zhu, Y. Skourski, D. E. Graf, V. Sechovský, M. Vališka, G. G. Lonzarich, F. M. Grosche, and A. G. Eaton. A Quantum Critical Line Bounds the High Field Metamagnetic Transition Surface in UTe2. *Physical Review X*, 15(2):021019, 2025. doi:10.1103/physrevx.15.021019.
